# Book Review

**Published:** 1908-09

**Authors:** 


**Green's Encyclopædia and Dictionary of Medicine and Surgery.** Vol. VI.—*Lumbar Region—Nephrotomy.* Edinburgh: Wm. Green & Sons. 1907.—This volume contains forty-eight articles of more than a thousand words, that on the lungs reaching sixty-nine pages. We have perused the article on malaria, and find that it contains a record of all the newest work on the subject. That on tuberculosis is written by Dr. R. W. Philip, and contains the views of a noted specialist on everything relating to sanatoria and the sanatorium treatment of tubercular diseases. Like its predecessors, this volume may be relied upon to give the best information on the subjects included in its alphabetical scope.

**Green's Encyclopedia and Dictionary of Medicine and Surgery.** Vol. VIII. Physiology—Rhinolatia. Edinburgh: Wm. Green and Sons.—The monograph on physiology occupies nearly one hundred pages, and it deals at some length with the internal secretions, or hormones, their production and action. The supra-renal bodies, the pituitary body, the thyroid, parathyroid, and other hormone-producing glands, appear at the present time to be endowed with various mystic qualities, which are presumed to be of great importance in the theories of toxic action and immunity. Dr. D. Noel Paton points out their importance in connection with the action of opsonins; but it seems well to suspend our judgment on many of the theoretical views of which Dr. Sajous is a distinguished exponent. Fifty-eight articles are of more than one thousand words in length. The volume contains 1037 subject headings, for the most part of the highest accuracy and value.

**A Chronology of Medical History.** By James Young, M.D. Pp. 91. Bristol: Edward Everard.—This chronological record of the famous names and important events connected with the history of medical science has been compiled from the larger works with much judgment and care. It begins with the first physician recorded in history, at the time of the fifth Egyptian Dynasty, 3500 B.C. The first known work on medicine, "The Ebers Papyrus," is dated 1550 B.C., and three hundred years later comes the time of Esculapius, 1250 B.C. Some two thousand years later came the prince of physicians—Avicenna, the Arabian Galen—the foremost representative of Moslem science, when the great universities of Bagdad, Damascus and Alexandria flourished, and when the cities of Cordova, Toledo and Seville were in their zenith as centres of learning. It is interesting to note that St. Bartholomew's Hospital was founded not long after, in 1123, and St. Andrew's University followed in 1411. The records continue to the close of the Victorian era, but the author wisely refrains from any description of the distinguished men who are happily alive at the present day.

**History of the Medical Society of the State of New York.** A centennial volume. By JAMES J. WALSH, M.D. Pp. 208. Published by the Society. 1907.—This volume is made up chiefly of excerpts from contemporary writings, by which the contemporaries are allowed, as far as possible, to tell their own tale. Historical accuracy has gained much by this method, but the author has carefully edited the various abstracts to make the story complete.

**Contributions to the Science of Medicine and Surgery.** By the Faculty, in celebration of the 25th Anniversary, 1882-1907, of the Founding of the New York Post-Graduate Medical School and Hospital. 1908. HENRY T. BROOKS, M.D., Editor.—This elegant volume, of nearly five hundred well printed pages, consists of papers on a great variety of subjects, such as are usually dealt with in post-graduate teaching. It shows how post-graduate opportunities have developed in the United States within the last quarter of a century. Clearly it will be difficult to surpass these post-graduate schools for real, earnest, enthusiastic work and development of technique. The obituary notice of the founder, and only president, of the Post-Graduate Medical School of New York is a fitting preface to the valuable mass of original work described in the pages which follow.

**Fevers in the Tropics.** By LEONARD ROGERS, M.D., B.S., F.R.C.P., F.R.C.S. Pp. 343. London: Henry Frowde and Hodder and Stoughton. 1908.—The subject of this volume is one of great importance to those resident in the tropics, and we have no doubt that the book will be heartily welcomed on the other side of the equator. But in Britain also it will be widely referred to, for in addition to descriptive detail, it contains the records of a large amount of original observation. Professor Rogers should be heartily congratulated upon the issue of so valuable a publication. After an account of the evolution of our knowledge of Indian fevers, and of the technics of blood examinations, the fevers of long duration are considered. These include kala-azar, trypanosomiasis and sleeping sickness, typhoid and paratyphoid, relapsing and African tick fevers, Malta or undulant fever, the pre-suppuration stage of amœbic hepatitis, epidemic dropsy, and some unclassified long fevers. Tropical typhoid presents many characteristic features of interest, and Professor Rogers has written this in a way which makes it very useful for purposes of reference. The section on Malta fever contains all the recent work of the Commission. With regard to the agglutination test, a dilution of 1 in 80 is recommended as necessary for diagnosis of the condition. Under epidemic dropsy is described a condition in which œdema of the extremities, most marked in the feet and legs, but often extending up to the thigh and abdomen, is associated with low, remittent or intermittent fever, commencing without any rigor and marked diarrhœa. The fevers of short duration comprise malarial fevers, dengue, plague, yellow fever, heatstroke, sunstroke, typhus, cerebro-spinal fever, influenza and the exanthemata. There are eleven good plates, and a number of illustrations and charts.

**Traité du Paludism.** By A. LAVERAN. Second Edition. Pp. 622. Paris: Masson. 1907.—This treatise will be found of great value by those who need to refer to the source of some of the statements contained in our numerous English text-books on the subject. The illustrations are good, and the descriptions clear and concise. Every scientific worker will welcome heartily this second edition, and will be glad to know that, tardy though the recognition has been, Laveran's early discoveries are at last gaining their due amount of acknowledgment.

**Transactions of the College of Physicians of Philadelphia.** Vol. XXIX. Philadelphia: Printed for the College. 1907.—One of the most interesting and useful papers in this volume is that on "The Treatment of Chronic Diseases of the Heart," by Professor Theodore S. Schott, of Nauheim, who, in conjunction with his late brother, August Schott, was the founder of the Balneogymnastic treatment of chronic heart disease associated with the name of Nauheim. After a brief review of other mechanical methods, more particularly that of Oertel and the Swedish method (that of Zander), he describes how, by means of balneologic, as well as gymnastic treatment, the heart becomes stimulated to a more forcible and vigorous systole. The baths acting through the sensory nerves, and the gymnastics acting through the motor nerves, achieve a similar result, and a bed-ridden patient may be relieved and strengthened to such an extent that he may be able to conclude his treatment with mountain climbing if the heart muscles have really become strong enough to bear the increased strain without undue risk.

**Diphtheria in Practice.** By W. F. LITCHFIELD, M.B. Pp. 96. London: Baillière, Tindall & Cox. 1908.—Following the short introduction is a chapter on cause and prevention, a simple outline sound as far as it goes, but it is not enough to state that "it would not appear that faulty sanitation plays an important part in the spread of diphtheria," for drains and diphtheria have no interdependence whatever. Again, the disinfection of rooms, etc., by sulphur is recommended as though it was our chief and best method, a mere passing note being accorded to the formalin spray. In the next chapter, on diagnosis, the value of clinical symptoms is emphasised, due regard being paid to the necessity for bacteriological examination in cases leaving room for doubt. But no mention is made of the use of direct cover slip examination of the suspected secretion, whereby valuable time may be saved in many cases. There is a truly colonial flavour in the remark that by the measures described the majority of cases "can be spotted right off." But farther on we find no clear directions for differentiating laryngismus stridulus and catarrhal laryngitis from diphtheria. We should expect clearer directions for the time that should intervene between the swabbing with antiseptics and taking a culture. The description of the various forms of the diphtheria bacillus is quite inadequate for a monograph. Good and very practical chapters on tracheotomy, intubation and on feeding will be found useful. The teaching is sound and practical, and much information of value in practice will be found in the small space the author has allowed himself.

**Third Annual Report of the Henry Phipps Institute for the study, treatment and prevention of Tuberculosis.** Pp. 410. Philadelphia. 1907.—This very elaborate Report gives an account of the work of the third year of the Institute; also a continuation of the report on the Maragliano serum treatment, a statistical study of the influence of the Henry Phipps Institute on the death-rate from tuberculosis in Philadelphia, and a report of some of the scientific work done by members of the staff of the Institute during the year.

It is disappointing to find that the serum treatment has been of no practical value, according to the present method of using it. It is hoped that some better ways of using what should be a successful method may be discovered.

**Some Successful Prescriptions.** By A. HERBERT HART, M.D. Pp. 17. London: John Bale, Sons and Danielsson Ltd. 1907.—The author wrote these on Monday, after the evening surgery. He might go on in this way for a whole year, but we fail to see the utility of a prescription for "skin diseases" and one for "malingerers." There are fifteen prescriptions; there is nothing new in them, and whatever is good is too familiar to be worthy of publication in a booklet.

**The Prevention of Infectious Diseases.** By JOHN C. M'VAIL, M.D., D.P.H. Pp. xv., 290. London: Macmillan & Co. Ltd. 1907.—This work comprises the Lane Lectures delivered at Cooper Medical College, San Francisco, in 1906. They are quite excellent, and reflect the experience and good judgment of the author on every page. They deserve to be read and re-read by all interested in public health who have passed beyond the student stage.

